# Cost-effectiveness analysis of durvalumab plus chemotherapy as first-line treatment for biliary tract cancer

**DOI:** 10.3389/fpubh.2023.1046424

**Published:** 2023-02-10

**Authors:** Zhuo-miao Ye, Zhe Xu, Huan Li, Qian Li

**Affiliations:** ^1^Department of Oncology, Xiangya Hospital, Central South University, Changsha, Hunan, China; ^2^Department of Pharmacy, Xiangya Hospital, Central South University, Changsha, Hunan, China; ^3^The Affiliated Changsha Central Hospital, Department of Infection Diseases, Hengyang Medical School, University of South China, Hengyang, Hunan, China

**Keywords:** cost-effectiveness analysis, durvalumab, biliary tract cancer, chemotherapy, Markov model

## Abstract

**Objective:**

The TOPAZ-1 trial reported a significant survival benefit of durvalumab in combination with chemotherapy for the first-line treatment of biliary tract cancer (BTC). However, no studies have evaluated the economics of this treatment option. The aim of this study was to assess the cost effectiveness of durvalumab plus chemotherapy compared to placebo plus chemotherapy from the perspective of US and Chinese payers.

**Methods:**

Based on clinical data from the TOPAZ-1 trial, a Markov model was developed to simulate 10-year life expectancy and total healthcare costs for patients with BTC. The treatment group received durvalumab in combination with chemotherapy and the control group received placebo plus chemotherapy. The primary outcomes analyzed included quality-adjusted life years (QALYs) and incremental cost-effectiveness ratios (ICERs). Uncertainty in the analysis results was assessed by sensitivity analysis.

**Results:**

For US payers, the placebo plus chemotherapy group had a total cost of $56,157.05 and a utility of 1.10 QALYs, while the durvalumab plus chemotherapy group had a total cost of $217,069.25, a utility of 1.52 QALYs, resulting in an ICER of $381,864.39/QALY. For Chinese payers, the ICER of durvalumab plus chemotherapy group was $367,608.51/QALY. Sensitivity analysis showed that the analysis was most sensitive to the price of durvalumab. For US and Chinese payers, under the respective willing to pay thresholds, the likelihood of the durvalumab plus chemotherapy arm being cost-effective was 0%.

**Conclusions:**

Both in China and in the US, durvalumab in combination with chemotherapy is not a cost-effective option for the first-line treatment of BTC compared with chemotherapy.

## Key points

• Our study provided the first assessment of the cost-effectiveness of durvalumab plus chemotherapy for the first-line treatment of advanced biliary tract cancer and showed that the regimen was not cost-effective for both US and Chinese payers. Further price reductions for durvalumab were needed.

## 1. Introduction

Biliary tract cancers (BTCs) includes Intrahepatic, perihilar, distal cholangiocarcinoma (based on the anatomical location of the biliary tract) and gallbladder carcinoma (1). Perihilarcholangio-carcinoma (pCCA) accounts for the highest proportion (50–60%), followed by Intrahepatic carcinoma Cholangiocarcinoma (iCCA) (20–30%) (2). Cholangiocarcinomas (CCAs) occur in 2.8–3.3 per 100,000 Asians and Hispanics (3). The iCCA mortality rate rose from 2.15 per 100,000 in 2009 to 2.95 per 100,000 in 2018, with an annual increase of 3.5% (4). The incidence of BTC is strongly associated with hepatitis C in US and European populations, whereas hepatitis B is significantly associated with the incidence of iCCA in Chinese and Korean populations (5, 6). In Asian countries, hepatolithiasis and gallbladder stones are risk factors for the high incidence of BTC, especially iCCA, and 70% to 90% of gallbladder cancer patients are secondary to chronic cholecystitis caused by stones (7). In addition, hepatobiliary fluke infection, primary sclerosing cholangitis, chronic inflammation with liver injury are also pathogenic factors (8–10). Cholangiocarcinoma has a poor prognosis, with a 5-year survival rate of about 10% (2). 75% of CCA patients are advanced at the time of diagnosis, and 70% of patients have disease recurrence after surgery, although surgery is the main treatment (11, 12). Chemotherapy is still the first-line treatment for advanced BTC. Since 2010, the ABC-02 trial in the United Kingdom established cisplatin plus gemcitabine (GP) as the first-line chemotherapy for advanced CCA. In this trial of 410 patients, gemcitabine plus cisplatin compared with gemcitabine alone, Improved median progression free survival (mPFS) (8.0 vs. 5.0 months) and median overall survival (mOS;11.7 vs. 8.1 months). Immune checkpoint proteins, which regulate the immune system, have the ability to recognize and destroy tumor cells. Among them, the immune checkpoint inhibitors (ICIs), including programmed cell death protein-1 (PD-1), programmed apoptosis ligand 1 (PD-L1) and cytotoxic T lymphocyte-associated antigen 4 (CTLA-4), inhibit antitumor immune responses in solid tumors (13, 14). BTC is a highly heterogeneous tumor caused by tumor gene mutations, which may be related to the expression of neoantigens (14). The biochemical environment of immunosuppression is generated by the tumor microenvironment (15, 16). BTC shows immunogenic characteristics in tumor microenvironment, and relevant studies have shown the clinical value of ICIs in BTC, such as durvalumab (17, 18). Durvalumab is a human IgG1 monoclonal antibody that selectively binds PD-L1 (18). Durvalumab previously showed promising efficacy in a phase 2 trial of the combination of gemcitabine and cisplatin, with an objective response rate of 72%, and its randomized, double-blind, phase 3 trial (TOPAZ-1; Clinicialtrials.gov number, NCT03875235), durvalumab plus chemotherapy significantly improved OS (24.9 vs. 10.4%) and objective response rate (26.7 vs. 18.7%) (17).

Despite the promising clinical applications of these two treatments, their high cost had attracted great attention. According to previous studies, the cost-effectiveness analysis of durvalumab was mostly performed in patients with small-cell lung cancer and non-small-cell lung cancer (19, 20). Studies of PD-1 inhibitors in BTCs were lacking. Therefore, in this study, we aimed to compare the cost-effectiveness of durvalumab combined with GP in advanced BTC from the perspective of healthcare payers in China and the United States (US).

## 2. Methods

### 2.1. Population

The basic medical data used in this economic evaluation referred to a double-blind, placebo-controlled, phase 3 global study (TOPAZ-1). The recruited patients were those with previously untreated disease that was unresectable or metastatic at initial diagnosis as well as those who developed recurrent disease more than 6 months after surgery with curative intent and more than 6 months after the completion of adjuvant therapy. This study included 424 patients and included the experimental group (198 patients) that has received durvalumab therapy and a control group (226 patients) that has received GP monotherapy.

### 2.2. The model's structure

Our analysis included 424 patients who have enrolled in the TOPAZ-1 trial as the target population. Based on the TOPAZ-1 trial, the Markov model was constructed for cost-effectiveness analysis of durvalumab as the first-line treatment for patients with BTCs. The model was built and run using Treeage Pro 2021 (Inc, Williamstown, MA, USA). This model has often been used by researchers for pharmacoeconomic analyses of advanced and metastatic cancer treatment (21, 22). The model included three health states: PFS, progressive disease (PD) and death. In the initial stage of the model, all patients are in an PFS state. As the treatment progressed, the patient either moved to another state or stays in this state. When the disease progressed, we assumed that the patients received chemotherapy (FOLFOX), immunotherapy (durvalumab), anti-angiogenesis inhibitor (regorafenib),Other therapy (Irinotecan plus capecitabine) as standard second-line treatment, as recommended by the Chinese Society of Clinical Oncology (CSCO) guidelines (version 2022) and National Comprehensive Cancer Network (NCCN) Guidelines for the diagnosis and treatment of primary BTCs (version 2022.2) (23). Notably, once a patient entered the PD state, they cannot return to the SD state; they either remained in the PD state or were transferred to the death state during the subsequent cycle. The specific transitions of each state in the model were shown in Supplementary Figure 1.

In the TOPAZ-1 clinical trial, the mOS in the experimental group was 12.8 months compared to 11.5 months in the control group, for a total study duration of no more than 2 years. However, Immunotherapy had a delayed effect and may continue to exert its beneficial effects beyond the treatment period; therefore, it should be analyzed using from long-term data to avoid inaccuracies and uncertainties in the results. Hence, with reference to the dosing cycle of the TOPAZ-1 clinical trial, we set the cycle of the Markov model to 21 days and the time horizon of 10 years to simulate the entire life course of the patient (24). Study endpoints included total cost, life years (LYs), quality-adjusted life years (QALYs) and incremental cost effectiveness ratios (ICERs). A half-circle correction was conducted to simulate the transfer process more accurately. This research was based on the perspective of Chinese and US payers, applying discount rates of 3% and 5% to costs and utilities, respectively (25). For US payers, we set the willing to pay (WTP) threshold to $150,000/QALY. For Chinese payers, according to the World Health Organization (WHO), ICER was acceptable if it was below three times the gross domestic product (GDP) per capita. We set the WTP threshold at three 3 times China's GDP per capita in 2021 (US $38334). The research methods followed the Consolidated Health Economic Evaluation Reporting Standards (CHEERS) (Supplementary Table 1) (26).

### 2.3. Clinical data input

The survival data of the experimental and control groups were presented using the Kaplan–Meier (KM) curve of the TOPAZ-1 clinical trial. The GetData Graph Digitizer (version 2.26; http://getdata-graph-digitizer.com/download.php) was used to extract the data points on the KM curve. R software was used to run the algorithm of Guyot et al. to reconstruct the extracted curve (27). We selected the best distribution from the exponential, weibull, gamma, log-normal, log-logistic and gompertz distributions to fit the reconstructed individual patient data (28). According to the Akaike Information Criterion (AIC) and Bayesian information Criterion (BIC), log-logistic and gamma distributions were selected to predict the long-term survival status of patients (Supplementary Figure 2). Ishak et al. have reported that in the process of fitting the parameter distribution to the survival model, lower AIC and BIC values provide objective criteria for the final selection of the distribution (29). The selection process for the distribution and goodness of fit is shown in Supplementary Table 2. The transition probability between the states of the Markov model was calculated using the method described by Liu et al. (30). This method reasonably corrects the time-dependent transition probability of a dynamic Markov model.

### 2.4. The utility and cost estimates

We were unable to obtain specific utility values for the patients with PFS and PD status. We used data from previously published studies as the health utility of BTCs patients in PFS and PD states (0.76 for PFS and 0.68 for PD) (31). To simplify the calculation, Grade 3 or higher adverse events (≥3 AEs) with the highest incidence difference between the durvalumab plus GP and GP groups were selected. Costs were converted based on 2021 US dollar exchange rates (USD 1.0 = CNY 6.34). We only consider the direct costs associated with medication, follow-up treatment, administration, laboratory tests and major ≥3 AEs according to the TOPAZ-1 trial. We obtained the latest prices of the drugs involved in the study through the sales prices of local hospitals or by consulting local drug suppliers. The upper and lower price limits of the drugs were determined by referring to all winning bids on the national pharmaceutical data platform (www.yaozh.com). For advanced BTCs, according to China's National Basic Medical Insurance, Industrial injury insurance and maternity insurance drug catalog (32), durvalumab could not be covered to partially reduce patient payments. We present the prices of the relevant drugs as costs both before and after health insurance coverage in Table 1. Except for the cost of ≥3 AEs as a one-time cost input model, the costs were calculated based on the dose used in the clinical trial and on a three-week cycle. As some of the costs referred to previously published literature, we used the consumer price index (CPI) inflation calculator to adjust these costs to 2022 prices (38).

**Table 1:** Basic parameters input to the model and the ranges of the sensitivity analyses.

**Variable**	**Baseline value**	**Range**		**Distribution**	**References**
		**Minimum**	**Maximum**		
Log-logistic OS survival model in durvalumab + chemotherapy group	Shape = 1.81; Scale = 13.55	Fixed in model		ND	Model fitting
Log-logistic OS survival model in chemotherapy group	Scale = 2.22; Scale = 11.68	Fixed in model		ND	Model fitting
Log-logistic PFS survival model in durvalumab + chemotherapy group	Scale = 2.19; Scale = 7.07	Fixed in model		ND	Model fitting
Gamma PFS survival model in chemotherapy group	Scale = 2.76; rate = 0.38	Fixed in model		ND	Model fitting
**Risk for main adverse events**					
**Durvalumab** + **chemotherapy**					
Neutrophil count decreased	0.207	0.1656	0.2484	Beta	(17)
Neutropenia	0.192	0.1536	0.2304	Beta	(17)
Anemia	0.189	0.1512	0.2268	Beta	(17)
Platelet count decreased	0.08	0.064	0.096	Beta	(17)
**Chemotherapy**					(17)
Neutrophil count decreased	0.254	0.2032	0.3048	Beta	(17)
Neutropenia	0.202	0.1616	0.2424	Beta	(17)
Anemia	0.187	0.1496	0.2244	Beta	(17)
Platelet count decreased	0.076	0.0608	0.0912	Beta	(17)
**Health utility scores**					
Utility of PFS	0.76	0.61	0.91	Beta	(33)
Utility of PD	0.68	0.54	0.82	Beta	(33)
**Drug costs in the US, $/per cycle**					
Gemcitabine	15.06	12.04	18.07	Gamma	CMS
Cisplatin	8.72	6.97	10.46	Gamma	CMS
Durvalumab	11,730	9,384	14,076	Gamma	CMS
Oxaliplatin	26.76	21.41	32.11	Gamma	CMS
Calcium Folinate (CF)	52.48	41.98	62.97	Gamma	CMS
Fluorouracil	18.57	14.86	22.29	Gamma	CMS
Irinotecan	35.88	28.70	43.05	Gamma	CMS
Capecitabine	180.6	144.48	216.72	Gamma	CMS
Regorafenib	21,546	17,236.8	25,855.2	Gamma	CMS
**Drug costs in China, $/per cycle**					
Gemcitabine	5.92	4.74	7.11	Gamma	b
Cisplatin	4.96	3.96	5.95	Gamma	b
Durvalumab	11,225.18	8,980.15	13,470.22	Gamma	b
Oxaliplatin	112.14	89.71	134.57	Gamma	b
Calcium Folinate (CF)	22.24	17.79	26.69	Gamma	b
Fluorouracil	140.97	112.77	169.16	Gamma	b
Irinotecan	547.28	437.83	656.74	Gamma	b
Capecitabine	43.2	11.94	99.73	Gamma	b
Regorafenib	1,495.21	1,196.17	1,794.25	Gamma	b
Laboratory_test/per cycle-First hospitalization	482.07	45.60	662.13	Gamma	b
Laboratory_test in PFS status	266.00	91.96	446.06	Gamma	a
Laboratory_test in PD status	390.57	142.19	626.12	Gamma	a
Imaging examination in first hospitalization	1,457.11	1,221.95	1,832.77	Gamma	a
Imaging examination in PFS status	246.91	11.75	622.57	Gamma	a
Imaging examination in PD status	466.62	246.83	1,832.77	Gamma	a
Bed fees	349.12	49.46	1,219.47	Gamma	a
Care costs	404.74	71.10	1,030.49	Gamma	a
**Expenditures on main AEs, $**					
Neutrophil count decreased	466	373	559	Gamma	(34)
Anemia	531	425	638	Gamma	(34)
Neutropenia	354	283	425	Gamma	(34)
Platelet count decreased	1,814	1,451	2,177	Gamma	(35)
**Disutility due to AEs**					
Leukopenia	−0.09	−0.072	−0.108	Beta	(36)
Anemia	−0.125	−0.100	−0.150	Beta	(36)
Neutropenia	−0.09	−0.072	−0.108	Beta	(36)
Thrombocytopenia	−0.20	−0.160	−0.240	Beta	(37)
**Risk for subsequent therapy**					
**Durvalumab** + **chemotherapy**					
Chemotherapy	0.417	0.334	0.500	Beta	(17)
Targeted Therapy	0.035	0.028	0.042	Beta	(17)
Immunotherapy	0.009	0.007	0.011	Beta	(17)
Other	0.044	0.035	0.053	Beta	(17)
**Chemotherapy**					(17)
Chemotherapy	0.479	0.383	0.575	Beta	(17)
Targeted Therapy	0.047	0.038	0.056	Beta	(17)
Immunotherapy	0.047	0.038	0.056	Beta	(17)
Other	0.081	0.065	0.097	Beta	(17)

^a^Based on real hospital data.

^b^Comprehensive pricing and range in conjunction with local hospital and Chinese pharmaceutical databases (https://www.yaozh.com).

CMS, Centers for Medicare & Medicaid Services (https://www.cms.gov/medicare/medicare-part-b-drug-average-sales-price/2022-asp-drug-pricing-file) 2022 ASP Drug Pricing Files.

PD-L1, Programmed cell death-Ligand 1; AEs, Adverse events; OS, Overall survival; PFS, Progression-free survival.

The drug dose was based on actual clinical trials. In the GP plus durvalumab group, the patients received 1,500 mg of durvalumab and gemcitabine (1,000 mg/m^2^) and cisplatin (25 mg/m^2^) once every 3 weeks. In the control group, the patients received gemcitabine (1,000 mg/m^2^) and cisplatin (25 mg/m^2^) once every 3 weeks. According to a report on the status of Chinese residents' nutrition and chronic diseases in 2020, the average weight of the adult Chinese population was 64.8 kg (39). However, considering the long progression of BTCs, most patients are likely to be middle-aged and older adults, and in the advanced stage of the disease, patients are likely to suffer from weight loss and other discomforts. Therefore, we assumed that the average weight of patients was 60 kg. The weight set would be used to calculate the drug dose per cycle for durvalumab. A total of 42.5% of patients in the durvalumab plus GP group and 49.4% in the GP group received subsequent treatments.

### 2.5. Sensitivity analyses

A one-way sensitivity analysis was carried out to explore the parameters that might affect the ICER and the extent to which they might do so. Each parameter was independently changed by assuming ±20% of the expected value to determine the obvious influence on decision-making. In the probabilistic sensitivity analysis (PSA), we chose appropriate distributions for the parameters relevant to the inclusion in the model, e.g., costs (adverse effects of drugs and treatments) were gamma and risks (AEs) and health utility scores (PFS, PD and AE) were beta distributions. All parameters fluctuated between the 95% confidence interval (CI) (40).

## 3. Results

### 3.1. Base-case analysis

**Figure 1:**
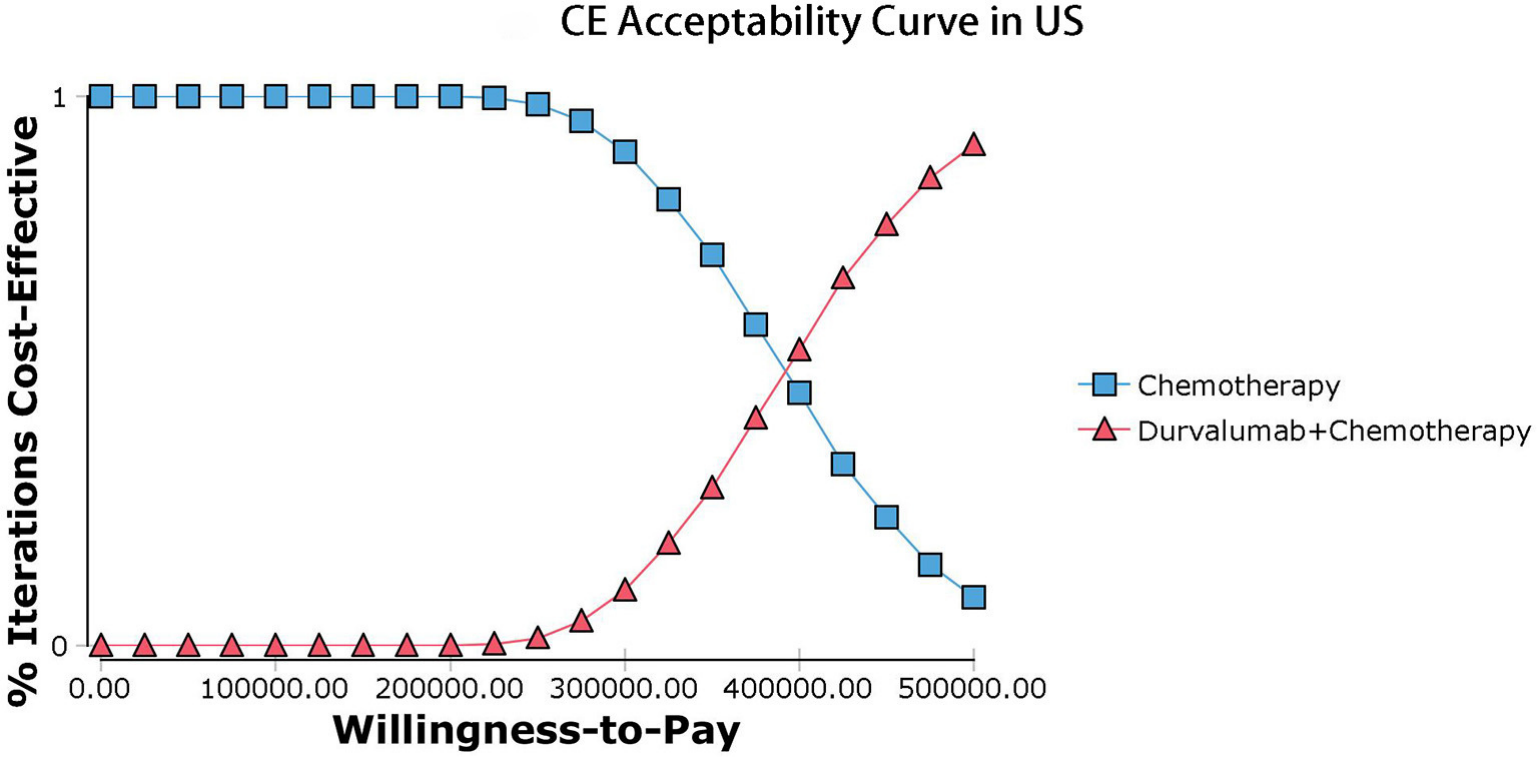
Acceptability curves in US.

**Figure 2:**
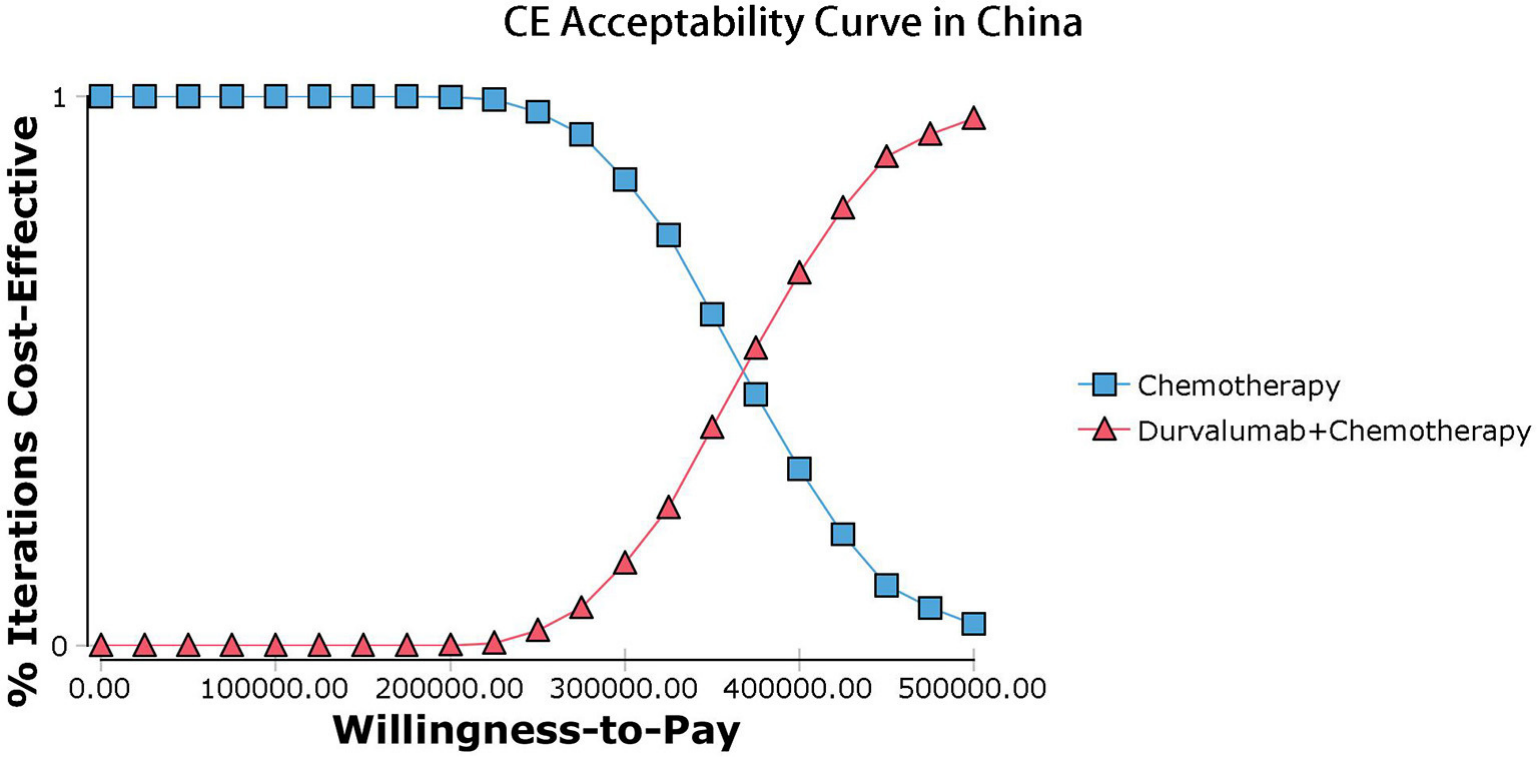
Acceptability curves in China.

Our model simulated the cost effectiveness of durvalumab or placebo combined with chemotherapy for 10 years in patients with advanced BTC. The results of the Base-Case Analysis were presented in Table 2. From the perspective of the US payers, the total cost incurred in the chemotherapy group was $56,157.05, with a health output of 1.10 QALYs and 1.66 LYs. The total cost incurred in the durvalumab plus chemotherapy group was $217,069.25 with a health output of 1.52 QALYs and 2.30 Lys (Figure 1). Therefore durvalumab plus chemotherapy incurred additional costs of $160,912.20 and 0.42 QALYs, resulting in ICERs of $381,864.39/QALY. From the perspective of the Chinese payers, compared to the chemotherapy group, the durvalumab plus chemotherapy group incurred an additional cost of $154,904.98, resulting in an ICER of 367,608.51 /QALY (Figure 2).

**Table 2:** Base-case analysis results.

**Strategies**	**Cost**	**Incr Cost**	**LYs**	**Incr LYs**	**ICER/ LYs**	**QALYs**	**Incr QALYs**	**ICER/QALYs**
**US payer perspective**								
Chemotherapy	56,157.05		1.66			1.10		
Durvalumab plus chemotherapy	217,069.25	160,912.20	2.30	0.64	251,818.78	1.52	0.42	381,864.39
**Chinese payer perspective**								
Chemotherapy	49,218.34		1.66			1.10		
Durvalumab plus chemotherapy	204,123.32	154,904.98	2.30	0.64	242,417.81	1.52	0.42	367,608.51

Incr Cost, Incremental cost; LYs, life-years; Incr LYs, Incremental life-years; QALYs, Quality-adjusted life-years; Incr QALYs, Incremental *Quality*-adjusted life-years.

### 3.2. Sensitivity analyses

**Figure 3:**
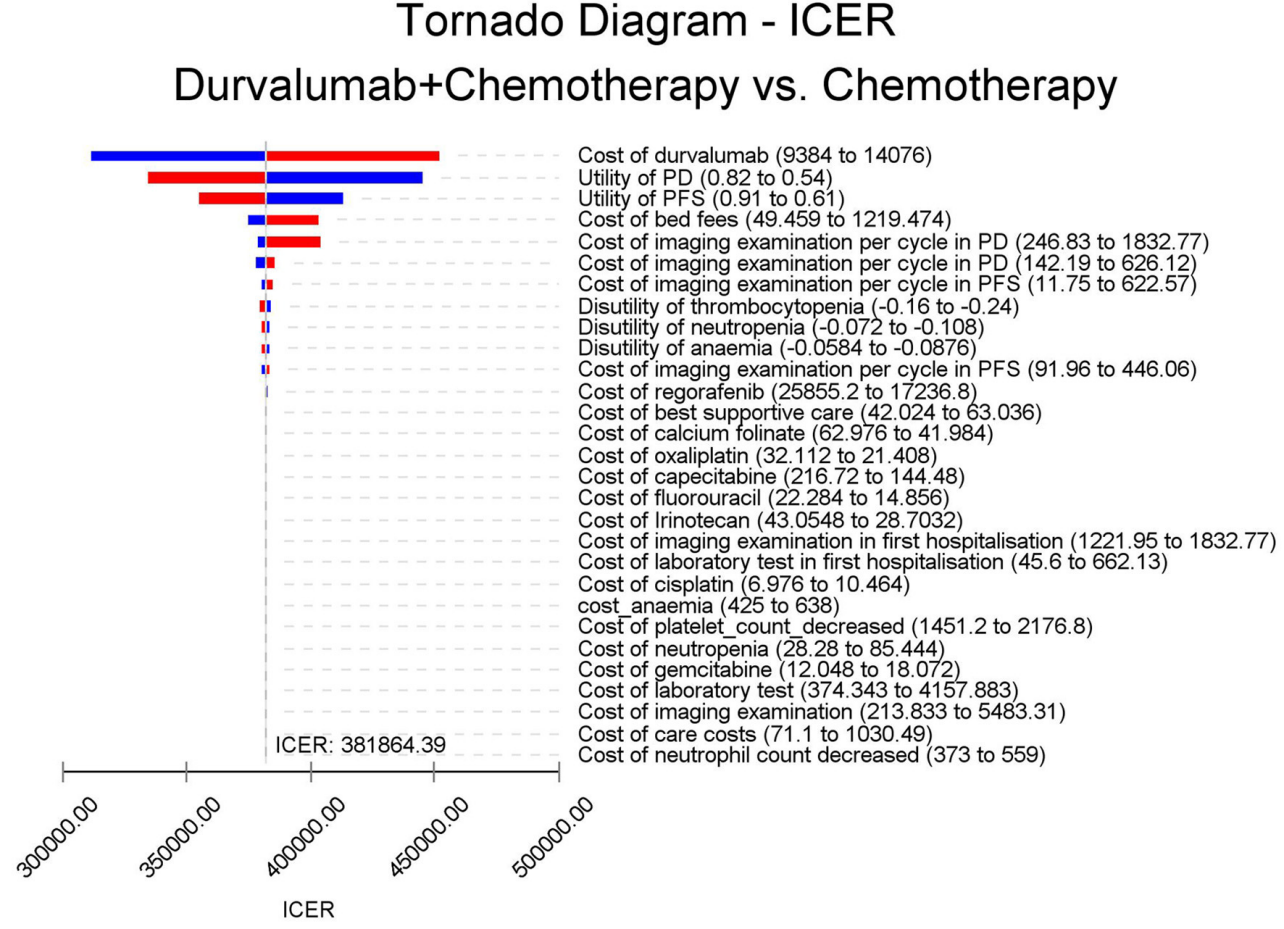
Tornado diagram for one-way sensitivity analysis in US.

**Figure 4:**
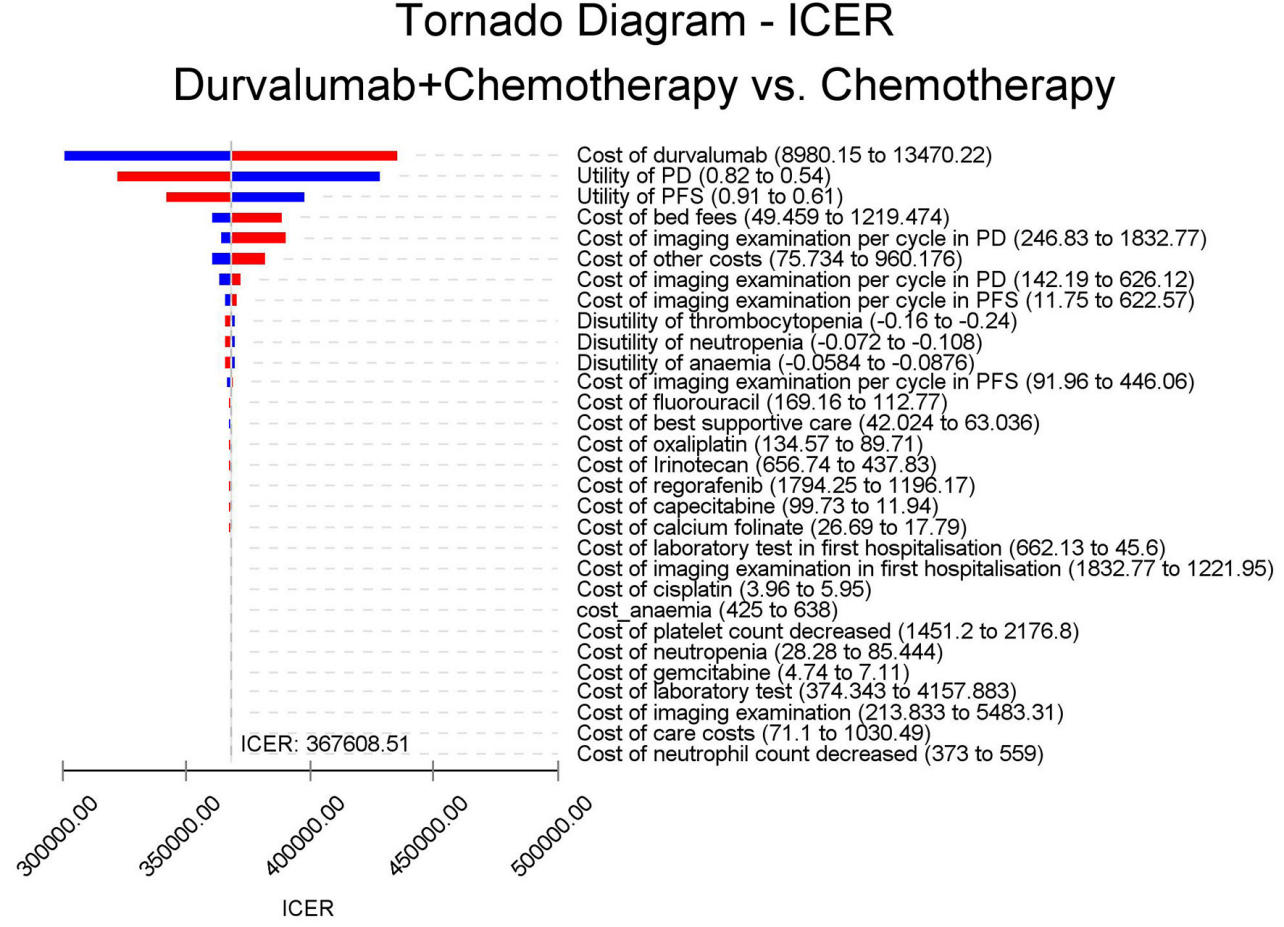
Tornado diagram for one-way sensitivity analysis in China.

**Figure 5:**
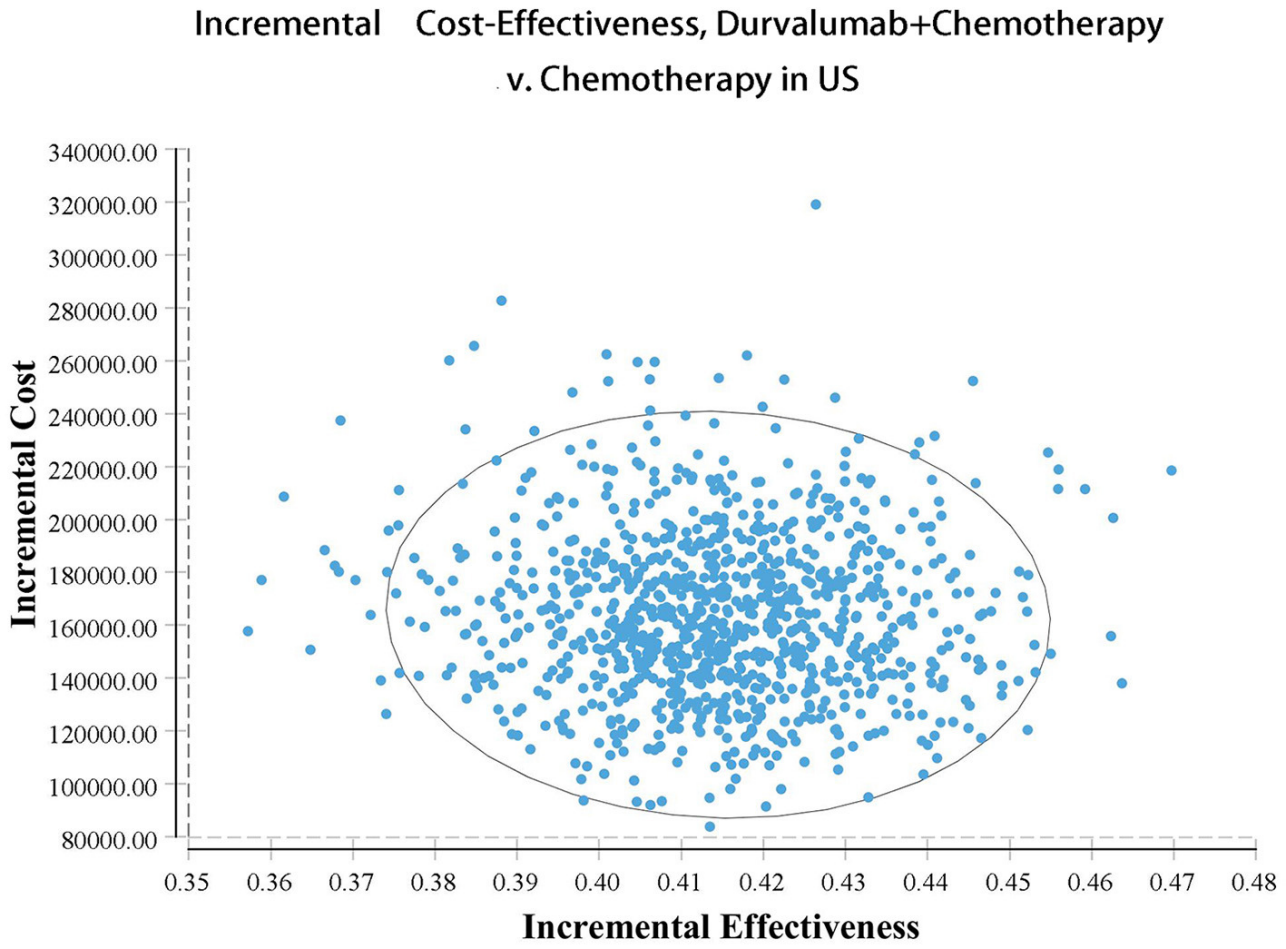
Incremental cost-effectiveness scatterplots in US.

**Figure 6:**
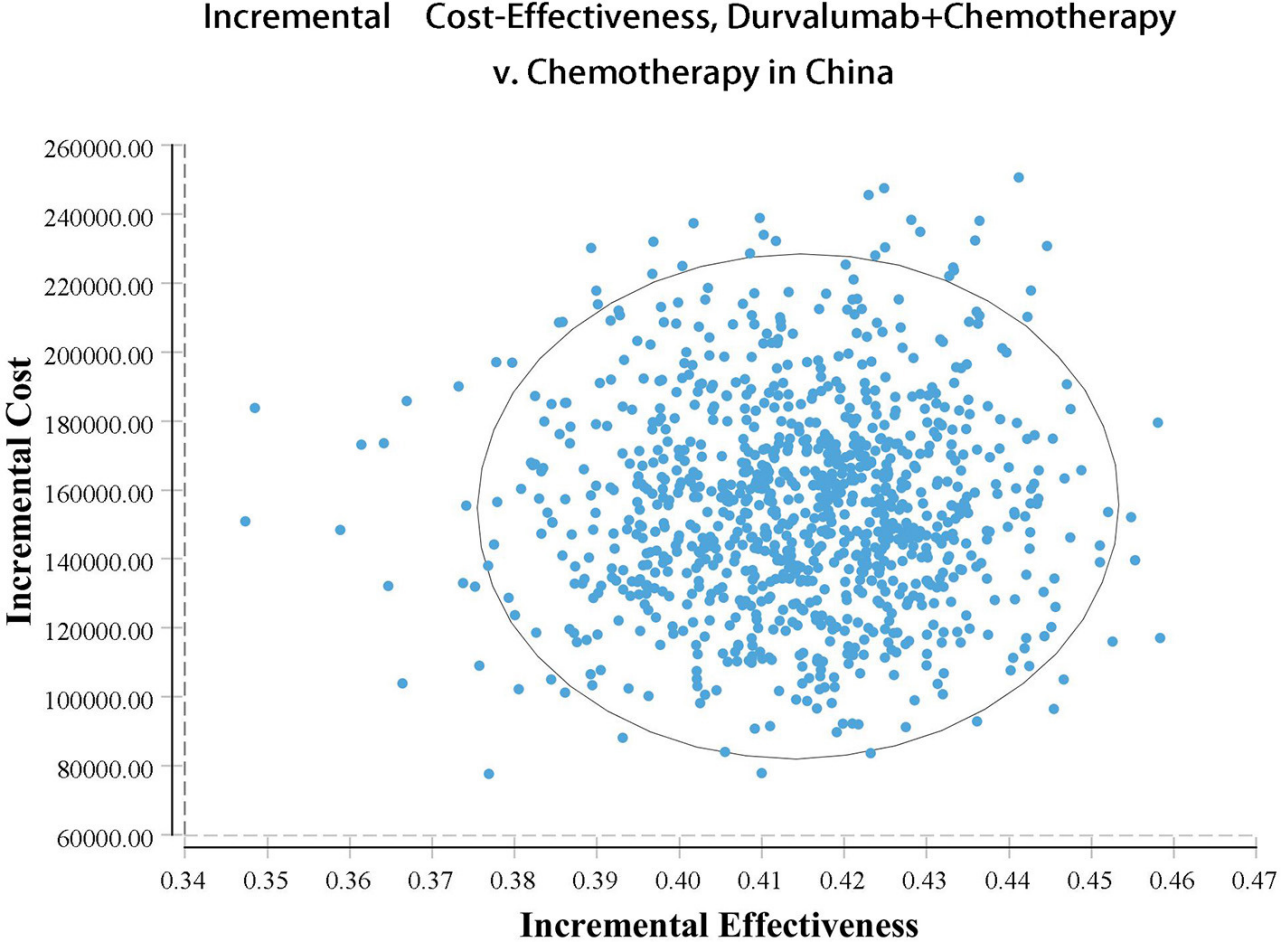
Incremental cost-effectiveness scatterplots in China.

The results of the deterministic sensitivity analysis were shown in the tornado diagram (Figures 3, 4). The main parameters that influenced the results of the analysis included the cost of durvalumab, the utility of PD and PFS status, with other parameters having minimal impact on the results. From the perspective of the US payers, when the price of durvalumab was varied at the given upper and lower limits, the ICER ranged from $311,653.61-$4452,075.17/QALY. However this was still well above the WTP threshold we set ($15,000/QALY). When the price of durvalumab was reduced by 67.4%, the ICER equaled $150,000/QALY. When the price of durvalumab was further reduced by 80.9%, the ICER equaled $100,000/QALY. The results of the PSA analysis showed a 0% probability of durvalumab plus chemotherapy regimens being cost effective at a WTP threshold of $150,000/QALY in the cost effectiveness acceptable curves (Figures 5, 6). Incremental cost scatter plots showed that the results of all Monte Carlo simulations were distributed above the WTP line, so that durvalumab plus chemotherapy was not cost-effective when all parameters vary within a given range. For Chinese payers, since the WTP thresholds in China was much lower than in the US, all parameters were equally not cost effective in the range of variation.

## 4. Discussion

Locally advanced BTC is too large and invasive of blood vessels to be surgically resected, and in the last decade, gemcitabine combined with cisplatin has usually been the first-line treatment option for such patients. However, chemotherapy alone has been ineffective, with limited patient benefit and a median OS of only 11.7 months (41). More recently, the TOPAZ-1 trial reported exciting clinical results with durvalumab in combination with chemotherapy for bile duct cancer. Durvalumab in combination with chemotherapy significantly improved OS and PFS in patients with BTC compared to standard chemotherapy, marking a milestone breakthrough in the treatment of BTC. The marketing application for a new indication for durvalumab in combination with chemotherapy for the first-line treatment of BTC has now been accepted by the Food and Drug Administration (FDA) and granted priority review. However, before clinicians can formally use this immune-combination chemotherapy regimen in clinical practice for patients with BTC, there are still some questions to be explored. As the most costly disease to treat in the United States, the cost of cancer treatment has increased significantly over the past decade and is still on an upward trend. The financial toxicity of ICI combination chemotherapy regimens, while improving efficacy, is seen as a negative consequence for cancer survivors (42). Excluding hospitalization expenses and toxicity, the direct cost of immunotherapy has exceeded the income of middle-class American families, and more than 1 in 3 patients cannot afford the financial toxicity of ICI, resulting in poorer quality of life and lower survival rates (43). Clinicians need to weigh the dual benefits of treatment cost and efficacy to develop the best treatment plan for patients of different economic levels. Therefore, to better facilitate the use of durvalumab in combination with chemotherapy regimens for bile duct cancer in clinical practice, it is necessary to evaluate its economics in terms of both cost and efficacy.

There was no study evaluating the economics of durvalumab in the treatment of BTC. Based on the latest clinical evidence from the TOPAZ-1 trial, our study constructed a Markov model to evaluate the cost-effectiveness of durvalumab combined with chemotherapy in the first-line treatment of BTC. The results of our analysis showed that the ICERs for durvalumab in combination with chemotherapy in the US and China were $426,301.52/QALY and $410,227.52/QALY, respectively. For US and Chinese payers, durvalumab plus chemotherapy did not offer a cost-effective advantage. The results of the sensitivity analysis showed that the price of durvalumab was a factor sensitive to the results of the analysis, followed by the utility of PD and PFS status. However, ICER far exceeds the WTP for US payers. All analyses showed no cost-effectiveness when all parameters were varied within a given interval. At current prices, the combination of durvalumab with chemotherapy for BTC is not economically advantageous, so further reductions in the cost of durvalumab are necessary. Further analysis of the price of durvalumab showed that durvalumab in combination with chemotherapy was only cost-effective when the price of durvalumab fell by 67.4% or more. If the WTP threshold of $100,000/QALY is used, the price of durvalumab needs to be reduced by more than 80.9%.

As the efficacy of durvalumab in combination with chemotherapy for the first-line treatment of BTC has only recently been revealed, there are still no studies evaluating the cost-effectiveness of this treatment option. Based on the current widespread use of durvalumab in lung cancer immunotherapy, several studies had evaluated the cost effectiveness of durvalumab for the treatment of lung cancer. Zhang et al. evaluated the cost effectiveness of durvalumab in combination with chemotherapy for the first-line treatment of small cell lung cancer from a US payer perspective (44). Zhang's analysis showed that the ICER of durvalumab plus chemotherapy was $355,448.86/QALY compared to the platinum-based chemotherapy regimen plus etoposide, so the regimen was not cost-effective. This result was consistent with the findings of Lin et al. although the ICER for durvalumab in combination with chemotherapy in Lin et al.'s analysis was $216,953/QALY (45). In addition, durvalumab in combination with chemotherapy was also not cost-effective for Chinese payers (46). It can therefore be seen that the cost of durvalumab in combination with chemotherapy needs to be further reduced for first-line treatment of small cell lung cancer. In the case of consolidation therapy after radiotherapy for non-small cell lung cancer, Han et al. showed that durvalumab was cost effective for US payers (47). The affordability of durvalumab was further validated in a microsimulation model of 2 million simulated patients conducted by Criss et al. (48) and could be extended to applicability to the US health care system. A study from Italy and others showed that the ICER of durvalumab in consolidation therapy after radiotherapy for non-small cell lung cancer exceeded the WTP threshold and that the official price of durvalumab needed to impose a discount (above 13%) to be cost-effective (49). These findings suggested that durvalumab plus chemotherapy for consolidation after radiotherapy for non-small cell lung cancer may be cost-effective in China and US, but not in Italy and other countries, and therefore geographical differences should be fully taken into account when conducting cost-effectiveness analyses. In addition, the current studies have reported no cost-effectiveness when this regimen was used as first-line treatment for small cell lung cancer. And this conclusion was also applicable to the first-line treatment of patients with BTC. Our study validated this in the first-line treatment of BTC, where durvalumab in combination with chemotherapy was not cost-effective in China and US. Two articles about cost-effectiveness analysis of BTC, but both of them were the comparison between chemotherapy regimen (Gemcitabine plus Cisplatin vs. Gemcitabine Alone). In Roth's study (50), gemcitabine monotherapy had the highest probability of being cost-effective until a willingness-to-pay of $60,000, Cost-effective until a willingness-to-pay of $60,000,after which the GP strategy had the highest probability. However, Tsukiyama's study (51) showed that combination therapy is less cost-effective than monotherapy for treating advanced BTC in Japan. In our study, compared with GP scheme, durvalumab+GP has better effect, but due to the high price of durvalumab, durvalumab+GP scheme is not cost-effective compared with GP scheme regardless of willingness to pay in China and USA.When the price of durvalumab is reduced, we can expect that durvalumab combined with GP regimen will be more suitable as a preferred option for patients with advanced BTC.

This study has a number of limitations. First, our model simulates patients from the TOPAZ-1 trial, which only published follow-up data for durvalumab combined with chemotherapy for about 2 years, and we digitally extracted OS and PFS data for durvalumab combined with chemotherapy and estimated them by parameter-specific survival distributions. Despite having a good good goodness of fit, its true long-term efficacy remains uncertain, which is subject to further refinement by subsequent follow-up data. Second, given that few studies have reported health utility in patients with BTC and that no specific utility data have been published from the TOPAZ-1 trial, we must make assumptions about health utility. We refer to previous studies reporting health utilities for patients with liver cancer and assume that the utilities for patients with BTC are consistent with them. This could lead to potential bias in the results of the analysis. The results of the analysis remain robust over the range of variation in utility. Thirdly, we only considered the impact of ≥3 AEs (increased costs and loss of utility), with 1–2 AEs being ignored, which are usually not or rarely intervened in clinical practice. In addition, ≥3 AEs with an incidence of < 5% were excluded from consideration, although sensitivity analyses showed that AEs had only a limited impact on the results of the analysis.

## 5. Conclusions

In comparison to chemotherapy, durvalumab plus chemotherapy is not considered cost-effective for first-line treatment of advanced BTC, either in China or in the United States. Further price reduction of durvalumab is necessary.

## Data availability statement

The original contributions presented in the study are included in the article/Supplementary material, further inquiries can be directed to the corresponding author.

## Author contributions

Z-mY and ZX had full access to all of the data in the study, take responsibility for the integrity of the data and the accuracy of the data analysis, concept and design, acquisition, analysis, or interpretation of data, drafting of the manuscript, and statistical analysis. HL and QL contributed to the critical revision of the manuscript for important intellectual content, obtained funding, administrative, technical, or material support, and supervision. All authors contributed to the article and approved the submitted version.

## Supplementary material

Supplementary Table 1 CHEERS checklist.

Supplementary Table 2 AIC and BIC scores of fitted distribution.

Supplementary Figure 1 Markov state transition probability diagram.

Supplementary Figure 2 Fitting of OS and PFS survival curves to different parameter survival distributions.

## References

[1] Rizvi S Gores GJ. Pathogenesis, diagnosis, and management of cholangiocarcinoma. Gastroenterology. (2013) 145:1215–29. 10.1053/j.gastro.2013.10.013 24140396 PMC3862291

[2] DeOliveira ML Cunningham SC Cameron JL. Cholangiocarcinoma: thirty-one-year experience with 564 patients at a single institution. Ann Surg. (2007) 245:755–62. 10.1097/01.sla.0000251366.62632.d3 17457168 PMC1877058

[3] Everhart JE Ruhl CE. Burden of digestive diseases in the United States Part III: Liver, biliary tract, and pancreas. Gastroenterology. (2009) 136:1134–44. 10.1053/j.gastro.2009.02.038 19245868

[4] Kim D Konyn P Cholankeril G Bonham CA Ahmed A. Trends in the mortality of biliary tract cancers based on their anatomical site in the United States From 2009 to 2018. Am J Gastroenterol. (2021) 116:1053–62. 10.14309/ajg.0000000000001151 33929380

[5] Donato F Gelatti U Tagger A Favret M Ribero ML Callea F. Intrahepatic cholangiocarcinoma and hepatitis C and B virus infection, alcohol intake, and hepatolithiasis: a case-control study in Italy. Cancer Causes Control. (2001) 12:959–64. 10.1023/A:1013747228572 11808716

[6] Yamamoto S Kubo S Hai S. Hepatitis C virus infection as a likely etiology of intrahepatic cholangiocarcinoma. Cancer Sci. (2004) 95:592–5. 10.1111/j.1349-7006.2004.tb02492.x 15245596 PMC11158843

[7] Hsing AW Bai Y Andreotti G Rashid A Deng J Chen J. Family history of gallstones and the risk of biliary tract cancer and gallstones: a population-based study in Shanghai, China. Int J Cancer. (2007) 121:832–8. 10.1002/ijc.22756 17450525 PMC2885776

[8] Tyson GL El-Serag HB. Risk factors for cholangiocarcinoma. Hepatology (Baltimore, Md). (2011) 54:173–84. 10.1002/hep.24351 21488076 PMC3125451

[9] Chapman MH Webster GJ Bannoo S Johnson GJ Wittmann J Pereira SP. Cholangiocarcinoma and dominant strictures in patients with primary sclerosing cholangitis: a 25-year single-centre experience. Eur J Gastroenterol Hepatol. (2012) 24:1051–8. 10.1097/MEG.0b013e3283554bbf 22653260 PMC3584158

[10] Ulrich F Pratschke J Pascher A. Long-term outcome of liver resection and transplantation for Caroli disease and syndrome. Ann Surg. (2008) 247:357–64. 10.1097/SLA.0b013e31815cca88 18216545

[11] Jeong S Cheng Q Huang L Wang J Sha M Tong Y. Risk stratification system to predict recurrence of intrahepatic cholangiocarcinoma after hepatic resection. BMC Cancer. (2017) 17:464. 10.1186/s12885-017-3464-5 28673346 PMC5496435

[12] Komaya K Ebata T Yokoyama Y Igami T Sugawara G Mizuno T. Recurrence after curative-intent resection of perihilar cholangiocarcinoma: analysis of a large cohort with a close postoperative follow-up approach. Surgery. (2018) 163:732–8. 10.1016/j.surg.2017.08.011 29336813

[13] Postow MA Callahan MK Wolchok JD. Immune Checkpoint Blockade in Cancer Therapy. J Clin Oncol. (2015) 33:1974–82. 10.1200/JCO.2014.59.4358 25605845 PMC4980573

[14] Chen W Hu Z Song J Wu Y Zhang B Zhang L. The state of therapy modalities in clinic for biliary tract cancer. Front Biosci (Landmark Ed). (2022) 27:185. 10.31083/j.fbl2706185 35748261

[15] Nakamura H Arai Y Totoki Y Shirota T Elzawahry A Kato M. Genomic spectra of biliary tract cancer. Nat Genet. (2015) 47:1003–10. 10.1038/ng.3375 26258846

[16] Zhou G Sprengers D Mancham S Erkens R Boor PPC van Beek AA. Reduction of immunosuppressive tumor microenvironment in cholangiocarcinoma by ex vivo targeting immune checkpoint molecules. J Hepatol. (2019) 71:753–62. 10.1016/j.jhep.2019.05.026 31195061

[17] Oh D-Y Lee K-H Lee D-W Yoon J Kim T-Y Bang J-H. Gemcitabine and cisplatin plus durvalumab with or without tremelimumab in chemotherapy-naive patients with advanced biliary tract cancer: an open-label, single-centre, phase 2 study. Lancet Gastroenterol Hepatol. (2022) 7:522–32. 10.1016/S2468-1253(22)00043-7 35278356

[18] Stewart R Morrow M Hammond SA Mulgrew K Marcus D Poon E. Identification and characterization of MEDI4736, an antagonistic anti-PD-L1 monoclonal antibody. Cancer Immunol Res. (2015) 3:1052–62. 10.1158/2326-6066.CIR-14-0191 25943534

[19] Hussain S Klugarova J Klugar M. Cost-effectiveness analyses of durvalumab consolidation therapy versus no consolidation therapy after chemoradiotherapy in stage-III NSCLC. Lung Cancer (Amsterdam, Netherlands). (2022) 170:11–9. 10.1016/j.lungcan.2022.06.002 35691134

[20] Ionova Y Vuong W Sandoval O. Cost-effectiveness analysis of atezolizumab versus durvalumab as first-line treatment of extensive-stage small-cell lung cancer in the USA. Clin Drug Investig. (2022) 42:491–500. 10.1007/s40261-022-01157-3 35604530 PMC9188525

[21] Wan X Zhang Y Tan C Zeng X Peng L. First-line nivolumab plus ipilimumab vs sunitinib for metastatic renal cell carcinoma: a cost-effectiveness analysis. JAMA Oncol. (2019) 5:491–6. 10.1001/jamaoncol.2018.7086 30789633 PMC6459127

[22] Zhou K Zhou J Huang J. Cost-effectiveness analysis of atezolizumab plus chemotherapy in the first-line treatment of extensive-stage small-cell lung cancer. Lung cancer (Amsterdam, Netherlands). (2019) 130:1–4. 10.1016/j.lungcan.2019.01.019 30885327

[23] Oncology G. Guidelines of Chinese Society of Clinical Oncology(CSCO) *Hepatocellular Carcinoma* (2022).

[24] Ding D Hu H Liao M Shi Y She L Yao L. Cost-effectiveness analysis of atezolizumab plus chemotherapy in the first-line treatment of metastatic non-squamous non-small cell lung cancer. Adv Ther. (2020) 37:2116–26. 10.1007/s12325-020-01292-3 32193809

[25] Sanders GD Neumann PJ Basu A Brock DW Feeny D Krahn M. Recommendations for conduct, methodological practices, and reporting of cost-effectiveness analyses: second panel on cost-effectiveness in health and medicine. JAMA. (2016) 316:1093–103. 10.1001/jama.2016.12195 27623463

[26] Husereau D Drummond M Petrou S Carswell C Moher D Greenberg D. Consolidated Health Economic Evaluation Reporting Standards (CHEERS)—explanation and elaboration: a report of the ISPOR Health Economic Evaluation Publication Guidelines Good Reporting Practices Task Force. Value Health. (2013) 16:231–50. 10.1016/j.jval.2013.02.002 23538175

[27] Guyot P Ades AE Ouwens MJ Welton NJ. Enhanced secondary analysis of survival data: reconstructing the data from published Kaplan-Meier survival curves. BMC Med Res Methodol. (2012) 12:9. 10.1186/1471-2288-12-9 22297116 PMC3313891

[28] Sieg M Hartmann M Settmacher U Arefian H. Comparative cost-effectiveness of cabozantinib as second-line therapy for patients with advanced hepatocellular carcinoma in Germany and the United States. BMC Gastroenterol. (2020) 20:120. 10.1186/s12876-020-01241-y 32316925 PMC7171756

[29] Ishak KJ Kreif N Benedict A Muszbek N. Overview of parametric survival analysis for health-economic applications. Pharmacoeconomics. (2013) 31:663–75. 10.1007/s40273-013-0064-3 23673905

[30] Liu G Kang S Wang X Shang F. Cost-effectiveness analysis of atezolizumab versus chemotherapy as first-line treatment for metastatic non-small-cell lung cancer with different PD-L1 expression status. Front Oncol. (2021) 11:669195. 10.3389/fonc.2021.669195 33987103 PMC8111076

[31] Su D Wu B Shi L. Cost-effectiveness of atezolizumab plus bevacizumab vs sorafenib as first-line treatment of unresectable hepatocellular carcinoma. JAMA Netw Open. (2021) 4:e210037. 10.1001/jamanetworkopen.2021.0037 33625508 PMC7905498

[32] Security N. Notice on the issuance of the National Drug List for Basic Medical Insurance, Work Injury Insurance Maternity Insurance. (2021). Available online at: http://www.mohrss.gov.cn/xxgk2020/fdzdgknr/shbx_4216/gsbx/202112/t20211203_429397.html (accessed December 3, 2021).

[33] Zhao M Pan X Yin Y Hu H Wei J Bai Z. Cost-effectiveness analysis of five systemic treatments for unresectable hepatocellular carcinoma in China: an economic evaluation based on network meta-analysis. Front Public Health. (2022) 10:869960. 10.3389/fpubh.2022.869960 35493395 PMC9051228

[34] Yang F Fu Y Kumar A Chen M Si L Rojanasarot S. Cost-effectiveness analysis of camrelizumab in the second-line treatment for advanced or metastatic esophageal squamous cell carcinoma in China. Ann Transl Med. (2021) 9:1226. 10.21037/atm-21-1803 34532363 PMC8421963

[35] Wan N Zhang TT Hua SH Lu ZL Ji B Li LX. Cost-effectiveness analysis of pembrolizumab plus chemotherapy with PD-L1 test for the first-line treatment of NSCLC. Cancer Med. (2020) 9:1683–93. 10.1002/cam4.2793 31945265 PMC7050096

[36] Haddad R Cohen EEW Venkatachalam M Young K Singh P Shaw JW. Cost-effectiveness analysis of nivolumab for the treatment of squamous cell carcinoma of the head and neck in the United States. J Med Econ. (2020) 23:442–7. 10.1080/13696998.2020.1715414 31928375

[37] Guan H Liu G Xie F Sheng Y Shi L. Cost-effectiveness of Osimertinib as a second-line treatment in patients with EGFR-mutated advanced non-small cell lung cancer in China. Clin Ther. (2019) 41:2308–20 e11. 10.1016/j.clinthera.2019.09.008 31607559

[38] Centers for Medicare & Medicaid Services. Available online at: https://www.cms.gov/medicare/medicare-part-b-drug-average-sales-price/2021-asp-drug-pricing-files (2021).

[39] National Health and Family Planning Commission. Report on Nutrition and Chronic Disease Status of Chinese Residents. Beijing: National Health and Family Planning Commission (2020).

[40] Briggs AH Weinstein MC Fenwick EAL Karnon J Sculpher MJ Paltiel AD. Model parameter estimation and uncertainty analysis: a report of the ISPOR-SMDM Modeling Good Research Practices Task Force Working Group-6. Med Decis Making. (2012) 32:722–32. 10.1177/0272989X12458348 22990087

[41] Valle J Wasan H Palmer DH Cunningham D Anthoney A Maraveyas A. ABC-02 Trial Investigators. Cisplatin plus gemcitabine versus gemcitabine for biliary tract cancer. N Engl J Med. (2010) 362:1273–81. 10.1056/NEJMoa0908721 20375404

[42] Kirchhoff A Jones S. Financial toxicity in adolescent and young adult cancer survivors: proposed directions for future research. J Natl Cancer Inst. (2021) 113:948–50. 10.1093/jnci/djab014 33839777

[43] Azzani M Roslani AC Su TT. The perceived cancer-related financial hardship among patients and their families: a systematic review. Support Care Cancer. (2015) 23:889–98. 10.1007/s00520-014-2474-y 25337681

[44] Zhang L Hang Y Liu M Li N Cai H. First-line durvalumab plus platinum-etoposide versus platinum-etoposide for extensive-stage small-cell lung cancer: a cost-effectiveness analysis. Front Oncol. (2020) 10:602185. 10.3389/fonc.2020.602185 33344252 PMC7747765

[45] Lin S Luo S Gu D Li M Rao X Wang C. First-line durvalumab in addition to etoposide and platinum for extensive-stage small cell lung cancer: A U S-based cost-effectiveness. Anal Oncol. (2021) 26:e2013–e20. 10.1002/onco.13954 34431578 PMC8571771

[46] Liu G Kang S. Cost-effectiveness of adding durvalumab to first-line chemotherapy for extensive-stage small-cell lung cancer in China. Expert Rev Pharmacoecon Outcomes Res. (2022) 22:85–91. 10.1080/14737167.2021.1888717 33627014

[47] Han J Tian K Yang J Gong Y. Durvalumab vs placebo consolidation therapy after chemoradiotherapy in stage III non-small-cell lung cancer: An updated PACIFIC trial-based cost-effectiveness analysis. Lung Cancer (Amsterdam, Netherlands). (2020) 146:42–9. 10.1016/j.lungcan.2020.05.011 32512272

[48] Criss SD Mooradian MJ Sheehan DF Zubiri L Lumish MA Gainor JF. Cost-effectiveness and budgetary consequence analysis of durvalumab consolidation therapy vs no consolidation therapy after chemoradiotherapy in stage III non-small cell lung cancer in the context of the US health care system. JAMA Oncol. (2019) 5:358–65. 10.1001/jamaoncol.2018.5449 30543349 PMC6439842

[49] Armeni P Borsoi L Fornaro G Jommi C Grossi F Costa F. Cost-effectiveness and net monetary benefit of durvalumab consolidation therapy versus no consolidation therapy after chemoradiotherapy in stage III non-small cell lung cancer in the italian national health service. Clin Ther. (2020) 42:830–47. 10.1016/j.clinthera.2020.03.012 32354495

[50] Roth JA Carlson JJ. Cost-effectiveness of gemcitabine + cisplatin vs. gemcitabine monotherapy in advanced biliary tract cancer. J Gastrointest Cancer. (2012) 43:215–23. 10.1007/s12029-010-9242-0 21234709

[51] Tsukiyama I Ejiri M Yamamoto Y Nakao H Yoneda M Matsuura K. A cost-effectiveness analysis of gemcitabine plus cisplatin versus gemcitabine alone for treatment of advanced biliary tract cancer in Japan. J Gastrointest Cancer. (2017) 48:326–32. 10.1007/s12029-016-9885-6 27785685 PMC5660135

