# Supplementary material for: Cost-effectiveness analysis of durvalumab plus chemotherapy as first-line treatment for biliary tract cancer
Source: Front Public Health. 2023 Feb 10;11:1046424. doi: 10.3389/fpubh.2023.1046424 (PMC9950513; doi:10.3389/fpubh.2023.1046424)
Supplement: Supplementary Table 1 — CHEERS checklist. [file Data_Sheet_1.docx]

**eTable1.CHEERS Checklist：ltems to include when reporting economic evaluations of health interventions**

The **ISPOR CHEERS Task Force Report**, *Consolidated Health Economic Evaluation Reporting Standards(CHEERS)—Explanation and Elaboration: A Report of the ISPOR Health Economic Evaluations Publication Guidelines Good Reporting Practices Task Force*, provides examples and further discussion of the 24-item CHEERS Checklist and the CHEERS Statement. It may be accessed via the Value in Health or via the ISPOR Health Economic Evaluation Publication Guidelines - CHEERS: Good Reporting Practices webpage:

<http://www.ispor.org/TaskForces/EconomicPubGuidelines.asp>

| **Section** | **Item No** | **Recommendation** | **Reported**  **on page**  **No/line No** |
| --- | --- | --- | --- |
| **Title and Abstract** | | | |
| Title | 1 | Identify the study as an economic evaluation or use more specific terms such as “cost-effectiveness analysis", and describe the interventions compared. | 1/1-2 |
| Abstract | 2 | Provide a structured summary of objectives, perspective, setting, methods (including study design and inputs), results (including base case and uncertainty analyses), and conclusions. | 1/17-36 |
| **Introduction** | | | |
| Background and  Objectives | 3 | Provide an explicit statement of the broader context for the study.  Present the study question and its relevance for health policy or practice decisions. | 2/31-36 |
| **Methods** | | | |
| Target population and Subgroups | 4 | Describe characteristics of the base case population and subgroups analysed, including why they were chosen. | 2/38-3/2 |
| Setting and location | 5 | State relevant aspects of the system(s) in which the  decision(s) need(s) to be made. | 3/6-7 |
| Study perspective | 6 | Describe the perspective of the study and relate this to the costs being evaluated. | 3/29-30 |
| Comparators | 7 | Describe the interventions or strategies being compared and state why they were chosen. | 3/6-7 |
| Time horizion | 8 | State the time horizon(s) over which costs and  consequences are being evaluated and say why appropriate. | 3/29-30 |
| Discount rate | 9 | Report the choice of discount rate(s) used for costs and outcomes and say why appropriate. | 3/29-30 |
| Choice of heath outcomes | 10 | Describe what outcomes were used as the measure(s) of benefit in the evaluation and their relevance for the type of analysis performed. | 3/26-27 |
| Measurement of effectiveness | 11a | *Single study-based estimates:* Describe fully the design features of the single effectiveness study and why the single. | 3/5-7 |
|  | 11b | Synthesis-based estimates: Describe fully the methods used for identification of included studies and synthesis of clinical effectiveness data. | N/A |
| Measurement and valuation of preference based outcomes | 12 | If applicable, describe the population and methods used to elicit preferences for outcomes.  clinical effectiveness data. | N/A |
| Estimating resources  and costs | 13a | *Single study-based economic evaluation:* Describe  approaches used to estimate resource use  associated with the alternative interventions. Describe primary or secondary research methods for valuing each resource item in terms of its unit cost. Describe any adjustments made to approximate to opportunity costs. | N/A |
|  | 13b | Model- based economic evaluation: Describe approaches and data sources used to estimate resource use associated With model health states. Describe primary or secondary research methods for valuing each resource item in terms of its unit cost Describe any adjustments made to approximate to opportunity costs. | 3/5-12；  4/13-25 |
| Currency, price date,  and conversion | 14 | Report the dates of the estimated resource quantities and unit costs. Describe methods for adjusting estimated unit costs to the year of reported costs if necessary. Describe Methods for converting costs into a common currency base  and the exchange rate. | 4/24-25 |
| Choice of model | 15 | Describe and give reasons for the specific type of decision analytical model used. Providing a figure to show model structure is strongly recommended. | 3/9-12;  efigure1 |
| Assumptions | 16 | Describe all structural or other assumptions underpinning the decision-analytical model. | 3/ 12-19 |
| Analytical methods | 17 | Describe all analytical methods supporting the evaluation. This could include methods for dealing  with skewed, missing, or censored data; extrapolation methods; methods for pooling data; approaches to validate or make adjustments (such as half cycle corrections) to a model; and  methods for handling population heterogeneity and uncertainty. | 3/26-27  3/38-4/6- |
| **Results** | | | |
| Study parameters | 18 | Report the values, ranges, references, and, if used,  probability distributions for all parameters. Report reasons or sources for distributions used to represent uncertainty where appropriate. Providing a table to show the input values is strongly  recommended. | Table 1 |
| Incremental costs and outcomes | 19 | For each intervention, report mean values for the main categories of estimated costs and outcomes of interest, as well as mean differences between the comparator groups. If applicable, report incremental cost-effectiveness ratios. | 5/4-13 |
| Characterizing uncertainty | 20a | Single study- based economic evaluation: Describe the effects of sampling uncertainty for the estimated incremental cost and incremental  effectiveness parameters, together with the impact of methodological assumptions(such as discount rate, study perspective). | N/A |
|  | 20b | Model-based economic evaluation: Describe the effects on the results of uncertainty for all input parameters, and uncertainty related to the structure of the model and assumptions. | 5/15-28 |
| Characterizing heterogenecity | 21 | If applicable, report differences in costs, outcomes, or cost-effectiveness that can be explained by  variations between subgroups of patients with different baseline characteristics or other observed variability in effects that are not reducible  by more information. | N/A |
| **Discussion** | | | |
| Study findings, limitations,  generalizability, and  current knowledge | 22 | Summarise key study findings and describe how they support the conclusions reached. Discuss  limitations and the generalisability of the findings and how the findings fit with current knowledge. | 6/8-22,  7/1-15 |
| **Other** | | | |
| Source of funding | 23 | Describe how the study was funded and the role of the funder in the identification, design, conduct, and reporting of the analysis. Describe other non-monetary sources of support. | 9 |
| Conflicts of interest | 24 | Describe any potential for conflict of interest of study contributors in accordance with journal policy. In the absence of a journal policy, we recommend authors comply with International Committee of Medical Journal Editors recommendations. | 9 |

For consistency, the CHEERS Statement checklist format is based on the format of the CONSORT

statement checklist

The **ISPOR CHEERS Task Force Report** provides examples and further discussion of the 24-item

CHEERS Checklist and the CHEERS Statement. It may be accessed via the *Value in Health* link or via the ISPOR Health Economic Evaluation Publication Guidelines-CHEERS: Good Reporting Practices webpage: http://www.ispor.org/TaskForces/EconomicPubGuidelines.asp

The citation for the CHEERS Task Force Report is:

Husereau D, Drummond M, Petrou S, et al. Consolidated health economic evaluation reporting

standards (CHEERS]- - Explanation and elaboration: A report of the ISPOR health economic

evaluations publication guidelines good reporting practices task force. Value Health 2013;16:231-

50.

eTable2.AIC and BIC scores of fitted distribution.

| Distribution | OS of chemotherapy | | PFS of chemotherapy | | OS of durvalumab plus chemotherapy | | PFS of durvalumab plus chemotherapy | |
| --- | --- | --- | --- | --- | --- | --- | --- | --- |
|  | AIC | BIC | AIC | BIC | AIC | BIC | AIC | BIC |
| Exponential | 1583.31 | 1587.15 | 1545.24 | 1549.08 | 1474.31 | 1478.14 | 1622.92 | 1626.76 |
| Gamma | 1512.81 | 1520.49 | 1424.14 | 1431.82 | 1442.46 | 1450.13 | 1555.17 | 1562.83 |
| Weibull | 1513.89 | 1521.57 | 1432.78 | 1440.46 | 1445.64 | 1453.31 | 1566.68 | 1574.35 |
| Log-logistic | 1510.98 | 1518.67 | 1428.24 | 1435.92 | 1441.13 | 1448.79 | 1547.18 | 1554.84 |
| Log-normal | 1514.61 | 1522.29 | 1431.57 | 1439.25 | 1442.01 | 1449.67 | 1548.04 | 1555.71 |
| Gompertz | 1537.86 | 1545.54 | 1480.59 | 1488.27 | 1460.78 | 1468.45 | 1603.60 | 1611.27 |

Abbreviation: OS=Overall survival; PFS= Progression-free survival; AIC= Akaike information criterion; BIC= Bayesian information criterion.

eFigure1：Markov state transition probability diagram.


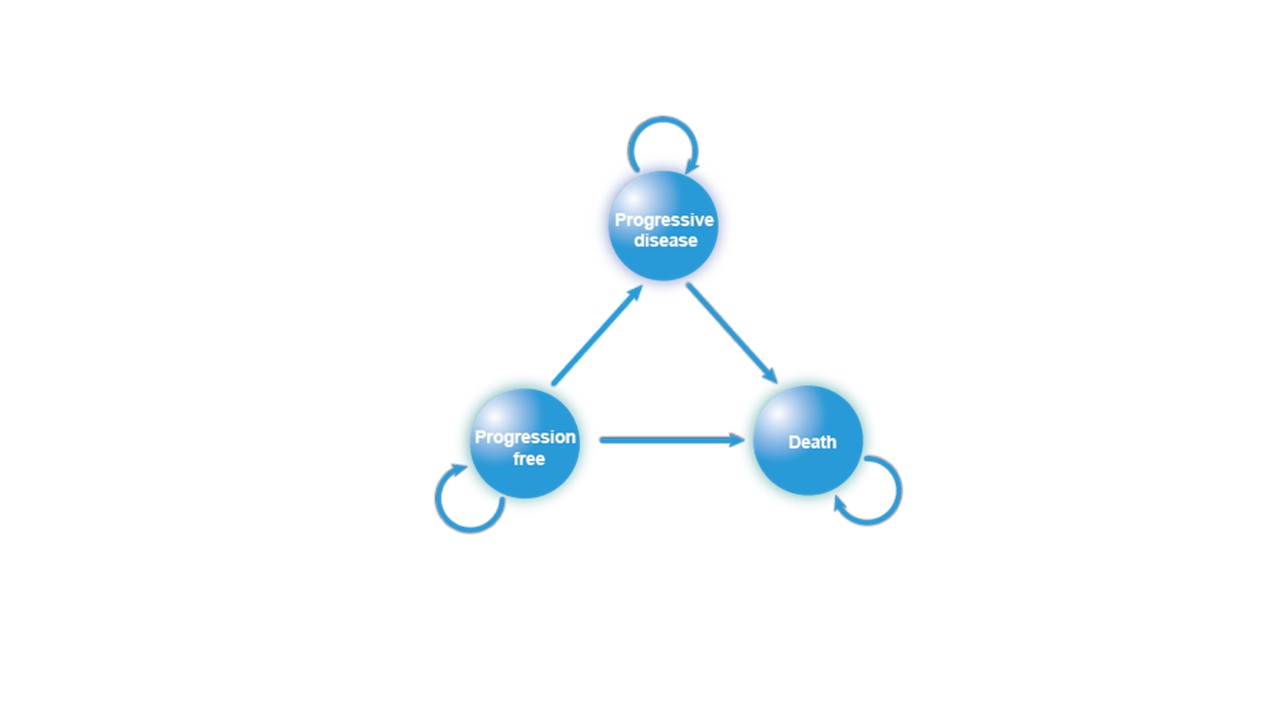


eFigure2 Fitting of OS and PFS survival curves to different parameter survival distributions


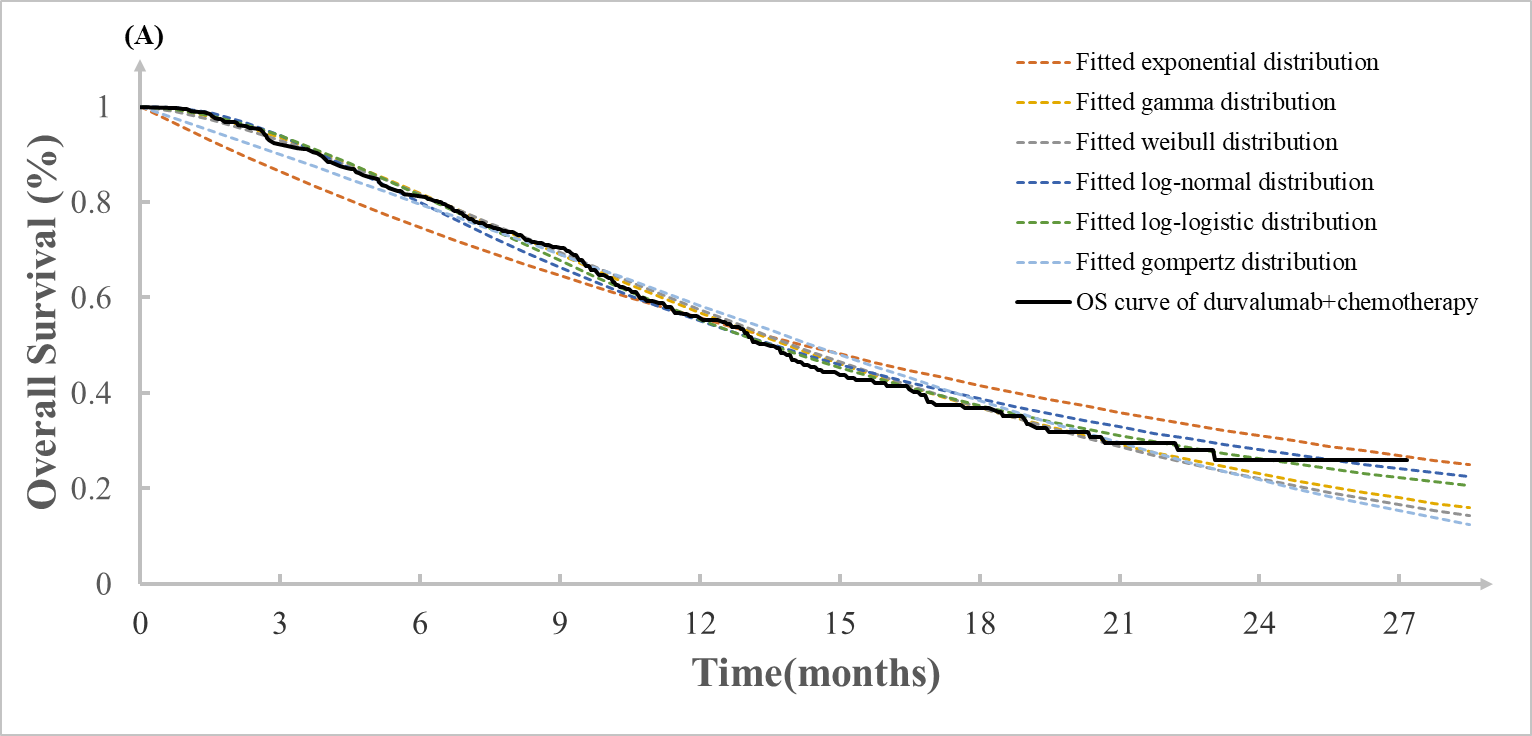


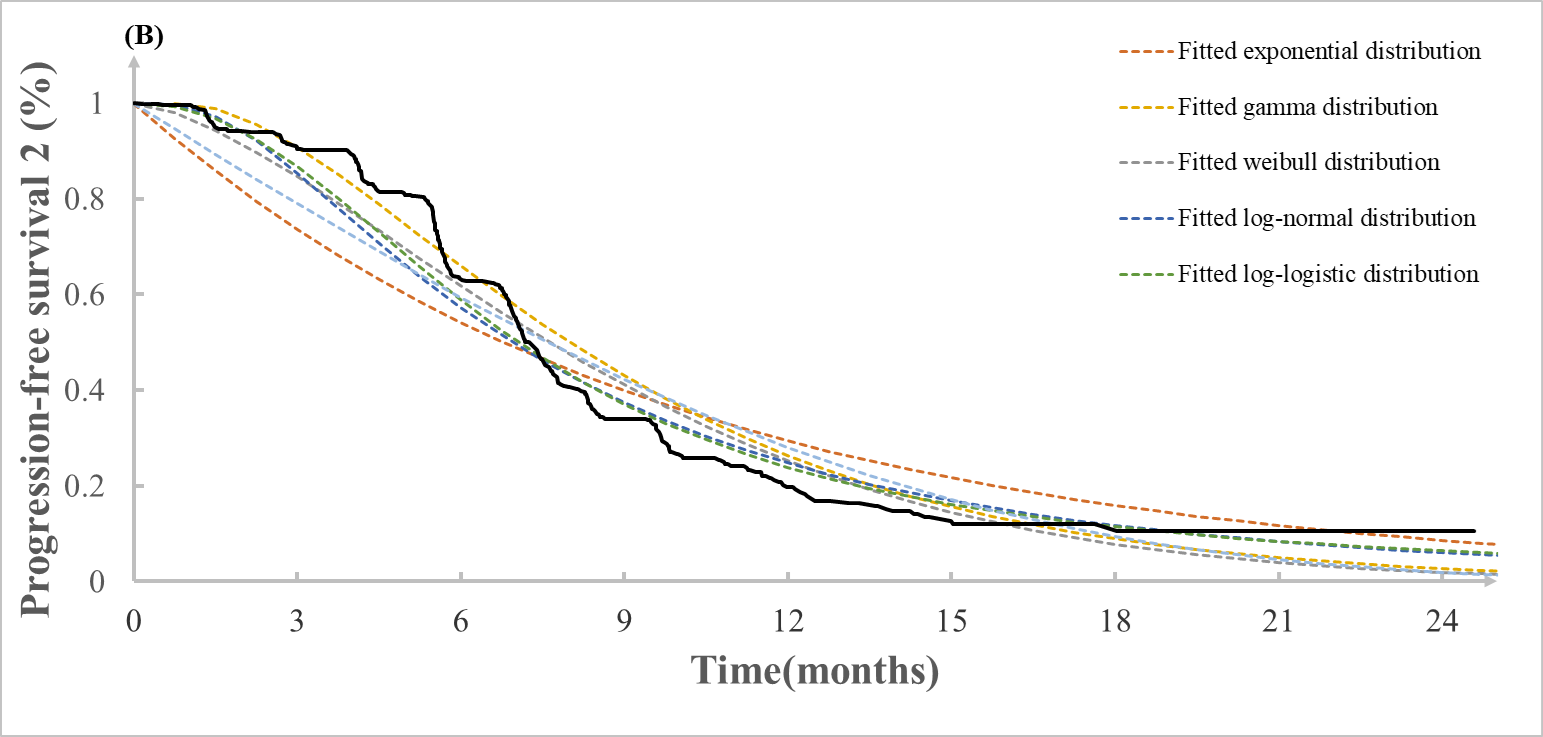


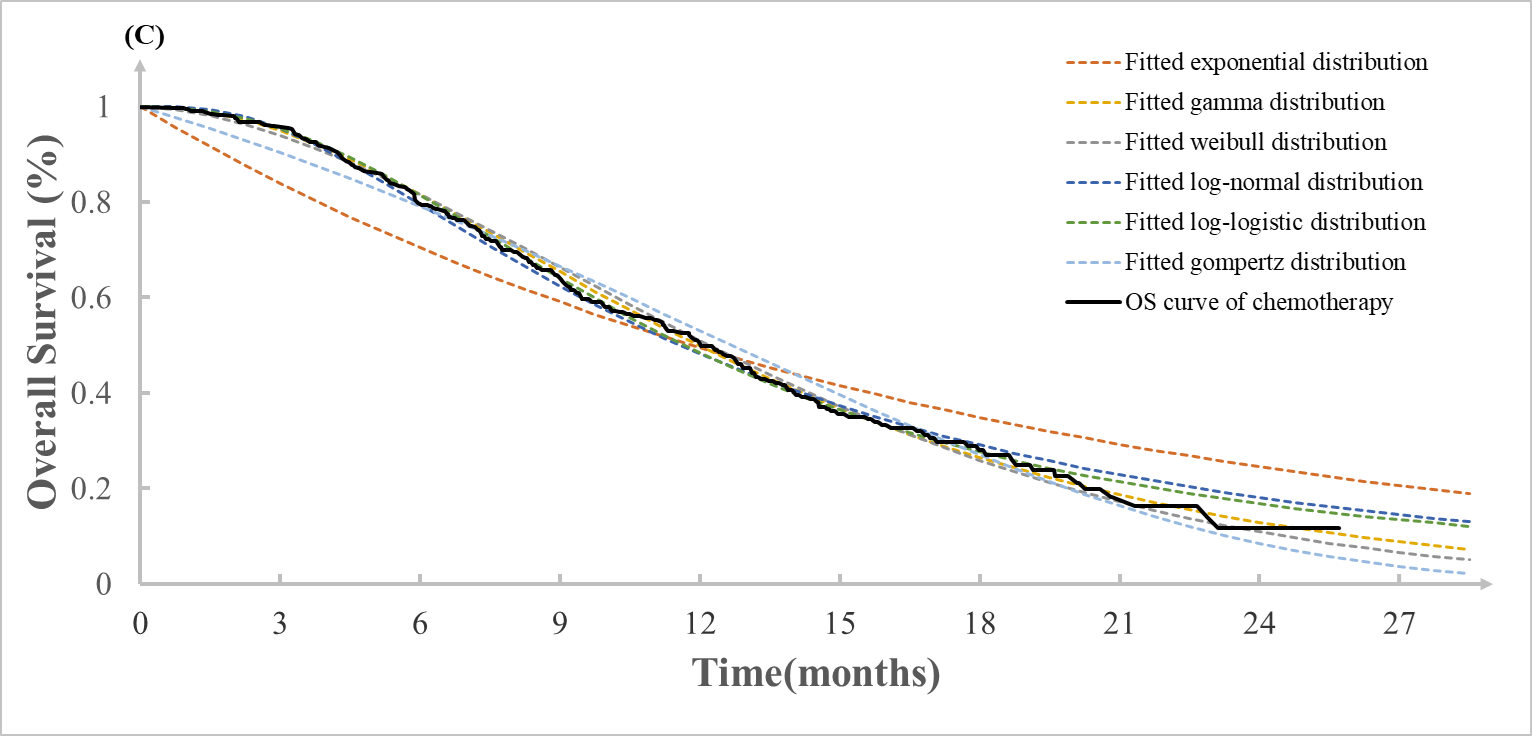


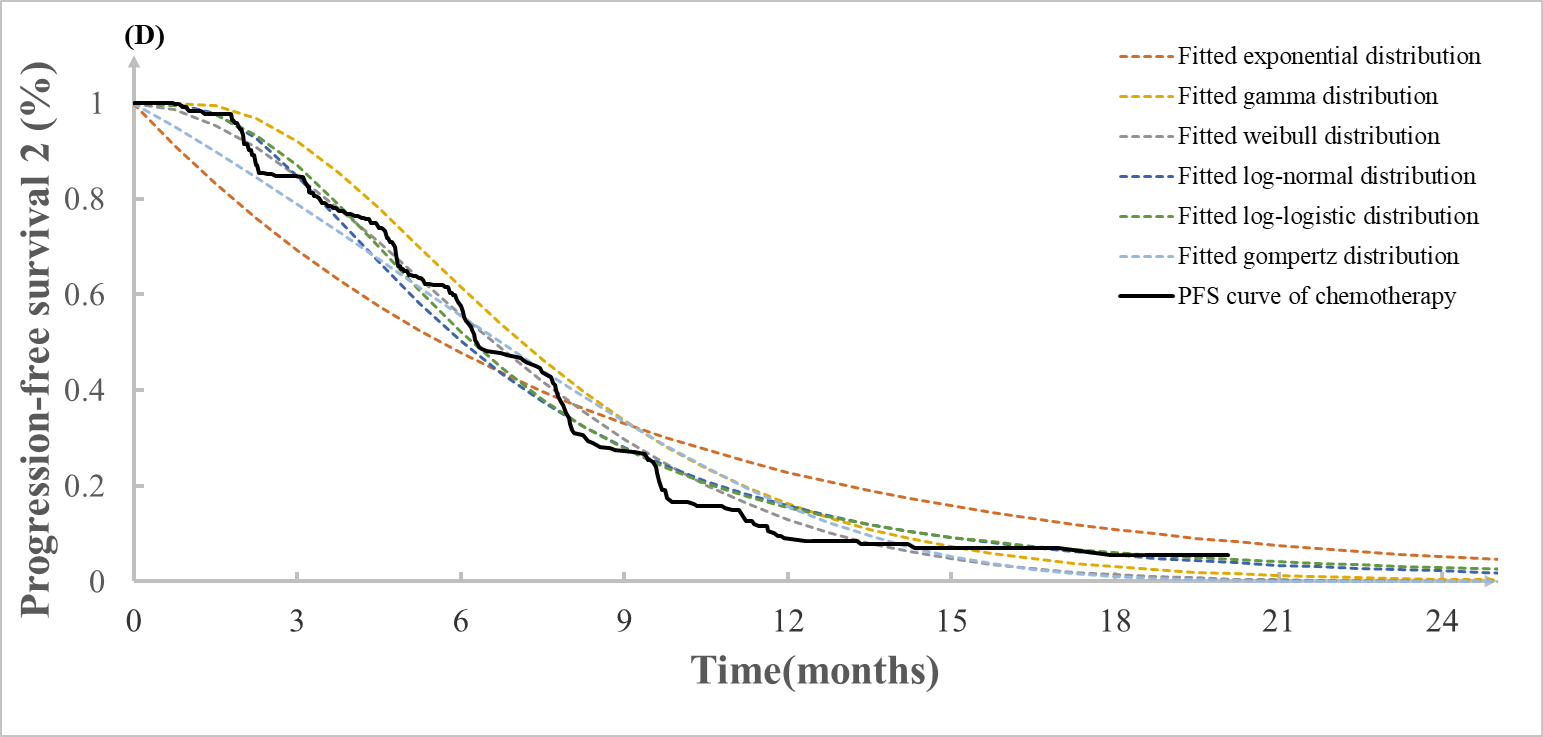


(A) OS-fitted curves in the durvalumab + chemotherapy group;

(B) PFS-fitted curves in the durvalumab + chemotherapy group;

(C) OS-fitted curves in the chemotherapy group;

(C) PFS-fitted curves in the chemotherapy group.

Abbreviations: OS= Overall Survival; PFS= Progression-Free-Survival; KM= Kaplan-Meier curves.
